# Global, regional, and national burdens of human papillomavirus-associated cervical cancer attributable to sexually acquired human papillomavirus infections among individuals aged 10-54 years from 1990 to 2021: findings from the Global Burden of Disease study 2021

**DOI:** 10.1093/sexmed/qfag045

**Published:** 2026-06-16

**Authors:** Pengkhun Nov, Duanyu Wang, Minghao Tan, Vicheth Virak, Xiaofang Zhou, Kun Liu, Youwen Zhu, Shishan Zhou, Hong Zhu

**Affiliations:** Department of Radiation Oncology and Oncology, LuangMe Hospital of University of Health Sciences, Phnom Penh 120110, Cambodia; Department of Radiotherapy, Zhujiang Hospital, Southern Medical University, Guangzhou, Guangdong Province 510282, China; Department of Oncology, Xiangya Hospital of Central South University, Changsha, Hunan Province 410008, China; Department of Gastrointestinal Surgery, Liuzhou Workers Hospital, Liuzhou, Guangxi Province 545005, China; Department of Cardiovascular Disease, Preah Ket Mealea Hospital, Phnom Penh 12203, Cambodia; Department of Oncology, Xiangya Hospital of Central South University, Changsha, Hunan Province 410008, China; Department of Oncology, Xiangya Hospital of Central South University, Changsha, Hunan Province 410008, China; Department of Oncology, Xiangya Hospital of Central South University, Changsha, Hunan Province 410008, China; Department of Oncology, Xiangya Hospital of Central South University, Changsha, Hunan Province 410008, China; Department of Oncology, Xiangya Hospital of Central South University, Changsha, Hunan Province 410008, China

**Keywords:** cervical cancer, Gbd, sexually acquired hpv infection, Hpv vaccine, autoregressive composite moving average

## Abstract

**Background:**

Human papillomavirus (HPV) infections that cause cervical cancer (CC) are transmitted through sexual contact. Certain sexual behaviors influence the risk of HPV acquisition and transmission.

**Aim:**

This study aimed to evaluate the global burden, temporal trends, and future projections of CC attributable to sexually acquired HPV infections among individuals aged 10-54 years to optimize prevention strategies.

**Methods:**

Data were extracted from the Global Burden of Disease (GBD) Study 2021. We analyzed deaths and disability-adjusted life years (DALYs) associated with CC linked to sexual transmission of HPV in the 10-54 age-group, stratified by age, Sociodemographic Index, and GBD region. The estimated annual percentage change was utilized to analyze trends from 1990 to 2021. Furthermore, Autoregressive Integrated Moving Average (ARIMA) models were applied to predict the disease burden trajectory through 2050.

**Outcomes:**

The primary outcomes assessed were the absolute counts and age-standardized rates (ASRs) of CC-related deaths and DALYs attributed to sexual transmission of HPV.

**Results:**

In 2021, there were 117 060 (95% uncertainty interval [UI], 106 690-128 467) CC deaths and 5 572 161 (95% UI, 5 057 538-6 116 191) DALYs globally among individuals aged 10-54 due to sexually acquired HPV infections. While the absolute numbers of deaths and DALYs increased by 23.9% and 23.1%, respectively compared to 1990, the age-standardized death and DALY rates significantly declined to 2.31 and 110.12 per 100 000. India and China bore the highest absolute burden, whereas Southern sub-Saharan Africa exhibited the highest standardized rates. Conversely, the Middle East and North Africa region reported the lowest burden. ARIMA models predict an increasing global burden in this demographic between 2021 and 2050.

**Clinical Implications:**

Targeted public health policies and increased HPV vaccination coverage are urgently required, particularly in high-burden, low- and middle-income regions, to counteract the projected rise in CC cases associated with sexual transmission of HPV.

**Strengths and Limitations:**

This study provides valuable insights into the HPV-associated burden of CC over 3 decades; however, it is limited by the GBD’s reliance on a standardized risk factor definition that may not capture all local behavioral nuances, potential underreporting in historical data due to undeveloped registry systems, reliance on modeling for data-scarce regions, and incomplete global data on HPV vaccine coverage.

**Conclusion:**

While ASRs of CC associated with sexually acquired HPV are declining, the absolute burden remains substantial and geographically disparate, highlighting the need for sustained global intervention.

## Introduction

Cervical cancer (CC) remains a major global public health challenge, with global cancer data for 2022 showing an expected 660 000 new cases and 350 000 deaths worldwide, particularly in low- and middle-income countries (LMICs), where preventive measures and therapies are often inadequate. This has resulted in a disproportionate rise in CC morbidity and mortality rates.^1^ As the fourth most prevalent cancer among women globally, CC is predominantly triggered by sustained infection with high-risk human papillomavirus (HPV) types, including HPV 18, 31, 33, 35, 39, 45, 51, 52, 56, 58, and 59. These HPV types are primarily transmitted through sexual contact. HPV is a viral particle encapsulating a circular double-stranded DNA genome that encodes a regulatory region, 2 structural proteins (L1 and L2), and several early proteins (E1-E7). The early proteins are involved in viral replication and contribute to the carcinogenic effects of high-risk HPV. L1, the major capsid protein, self-assembles into virus-like particles (VLPs), which induce HPV type-specific neutralizing antibodies and serve as the foundation for current HPV vaccines.^2^ Currently, the proven effective HPV vaccines include bivalent, quadrivalent, and 9-valent formulations. The bivalent and quadrivalent vaccines can prevent approximately 70% of HPV-related infections, whereas the 9-valent vaccine, which contains VLPs derived from HPV types 6, 11, 31, 33, 45, 52, and 58, is capable of preventing up to 90% of CCs caused by HPV.^3^

Sexual behaviors that influence the risk of HPV transmission represent 1 of the critical factors contributing to HPV transmission. Studies conducted in the United States, Ghana, Uganda, Iran, and sub-Saharan Africa^4–7^ show that certain behavioral patterns, such as early sexual debut, multiple sexual partners, or inconsistent condom use, remain relatively common, particularly among young women. In addition, in LMICs, medical infrastructure is often inadequate and vaccination programs are not widely implemented. These behavioral factors further increase the risk of HPV transmission and subsequent CC. Therefore, despite the availability of an effective HPV vaccine, the burden of preventable CC remains high, especially for young and middle-aged women (10-54 years of age) who are at the peak of their fertility and childbirth.

From 1990 to 2019, the global burden of HPV-associated CC exhibited mixed trends. While some regions observed declines in incidence and mortality due to advancements in screening and vaccination programs, other regions continued to experience increases in both metrics.^8^ Previous studies have demonstrated that young women are more likely to reduce their risk of CC following HPV vaccination.^9^ Nevertheless, the persistently high disease-related mortality in this demographic underscores the critical need for targeted interventions, such as comprehensive HPV vaccination coverage, accessible screening initiatives, and efficacious treatment strategies.

We hypothesized that the global burden of HPV-associated CC attributable to sexual transmission of HPV would exhibit substantial heterogeneity across regions and socioeconomic strata, with the highest burdens concentrated in LMICs where HPV vaccination coverage remains limited. Furthermore, we hypothesized that while some regions may show declining trends due to successful immunization programs, others, particularly those with delayed vaccine introduction, would continue to experience increasing or persistently high burdens, underscoring the critical need for targeted and equitable prevention strategies.

To address these gaps, this study was designed with the following primary objectives: (1) to quantify the global, regional, and national burdens of HPV-associated CC attributable to sexual transmission of HPV—as defined within the Global Burden of Disease (GBD) 2021 framework—among individuals aged 10-54 years from 1990 to 2021; (2) to evaluate temporal trends in these burdens across regions with varying levels of sociodemographic development; and (3) to project future trends up to 2030 and identify implications for targeted HPV vaccination and prevention policies. This study does not seek to establish causal attribution of CC to individual behaviors. Rather, it employs a population-attributable fraction approach within the GBD comparative risk assessment framework to estimate the burden associated with sexual transmission of HPV, thereby providing evidence to inform equitable and effective public health strategies. By clarifying the operationalization of the exposure and explicitly framing the research questions, this analysis aims to offer a clear and replicable foundation for understanding the preventable burden of HPV-associated CC and for guiding policy decisions toward the global elimination of this disease.

## Materials and methods

### Data source

Utilizing the latest epidemiological data and advanced standardized methodologies, the 2021 GBD study conducts a comprehensive evaluation of the impact caused by 371 different health conditions, injuries, and impairments, alongside 88 contributing factors, spanning 204 countries and regions. The findings of this study are available through the Global Health Data Exchange query tool (http://ghdx.healthdata.org/gbd-results-tool), divided into 27 geographic sectors.

Within the GBD 2021 framework, the risk factor analyzed in this study is defined as “sexual behaviors that influence the risk of HPV acquisition and transmission.” This definition is based on the comparative risk assessment approach used by GBD, which quantifies the population-attributable fraction of CC due to sexually transmitted HPV infections. It does not imply a value judgment of “safe” or “unsafe” practices but rather reflects the epidemiological evidence that HPV is sexually transmitted and that certain behavioral patterns modify transmission risk. For the purpose of this analysis, we adhere strictly to the GBD’s standardized risk factor definition without adding subjective qualifiers.

To assess the global burden of CC associated with sexual transmission of HPV in people aged 10-54 years, we extracted data from the Global Health Data Exchange, which includes CC death toll, the number of disability-adjusted life years (DALYs), corresponding age-standardization DALYs, age-standardized death rates (ASDRs), and other data (with the corresponding 95% uncertainty interval [UIs]).

Age-standardized rates (ASRs) are necessary and representative when comparing several individuals with different age structures or individuals at the same time. Data are disaggregated by age-group. The study also investigated time trends in the global burden of CC disease associated with sexual transmission of HPV in people aged 10-54 years from 1990 to 2021 using estimated annual percentage change (EAPC) values calculated by a linear regression model. EAPC was calculated using the method proposed by Hankey as a metric of the trend in ASR over a given time interval. ASR was simulated using a regression model: ln(ASR) = α + βx + ε, where α is the intercept term, β is the yearly change in the mortality rate per 100 000 population, *x* is the calendar year, and ε is the error term. The EAPC is formulated as 100 × (exp(β) - 1), and its 95% CI is derived similarly to that of the linear regression model.^10–12^

The Sociodemographic Index (SDI) functions as a comprehensive metric for evaluating socioeconomic, demographic, and developmental conditions in countries and regions. It incorporates factors such as per capita GDP, education levels, and fertility rates. The SDI is scaled from 0 to 1, with higher values indicating better socioeconomic conditions and lower values reflecting lesser development. This analysis classifies 204 nations and territories into 5 SDI categories: low, low-middle, middle, high-middle, and high. This classification enhances our understanding of how developmental factors influence health across various areas.

The GBD initiative utilizes the DisMod-MR 2.1 software, a Bayesian meta-regression tool, for estimating disease incidence with a methodical cascade process. Before analysis, adjustments are made to ensure data accuracy by disaggregating nonspecific age and sex data and employing a Meta-Regression-Bayesian, Regularized, Trimmed approach for consistent comparison across different study setups and definitions. Secondary and tertiary risk factors, selected based on World Cancer Research Fund guidelines, are analyzed using a comparative risk assessment model.^13^

### Statistical analysis

The study first presents the number of deaths and DALYs associated with sexual transmission of HPV in the 10-54 individual in 2021, as well as ASRs by age-group, SDI region, and GBD region. Then, the EAPC value of linear regression is used to analyze the time trend from 1990 to 2021. Spearman correlation analysis evaluated the relationship between EAPC and ASR. We then applied frontier analysis as a rigorous method to establish benchmarks for assessing the burden of CC disease caused by sexually transmitted HPV in different countries and regions. This approach facilitates comparative evaluation, comparing each entity to its best-performing counterpart, thereby identifying leading countries and establishing benchmarks that can guide and inspire others. Specifically, we calculated an “effective difference” indicator for each country and region, which quantifies the difference between the current disease burden of CC from sexually transmitted HPV and its potentially optimal level, adjusted for the SDI. This adjustment ensures that comparisons are fair, taking into account differences in socioeconomic and demographic conditions that can have a significant impact on disease outcomes. Finally, to predict future trends in the burden of CC associated with sexual transmission of HPV, we applied an Autoregressive Integrated Moving Average (ARIMA) model to make predictions. This technique requires transforming the time series data to a stationary state through differencing prior to analysis. The model is defined by 3 main parameters: p (autoregressive order), d (differencing order), and q (moving average order). These parameters were set based on analyses performed with tools like the autocorrelation function and the partial autocorrelation function. Forecasting was conducted using the forecast and tseries packages in R, facilitating both prediction and graphical display of the results. To ensure the reliability of our forecasts, various validation techniques were applied: checking the independence of forecast errors via the Ljung-Box Q-test, confirming the normal distribution of residuals with a mean of zero by the Shapiro–Wilk test, and evaluating the homoscedasticity of residuals through visual inspection and the Breusch–Pagan test. Statistical significance was determined with a *P*-value threshold of .05. Data construction, organization, and analysis were performed using R software.^13^

## Result

### The global burden of cervical cancer associated with sexual transmission of human papillomavirus in people aged 10-54 years, 2021

**Figure 1:**
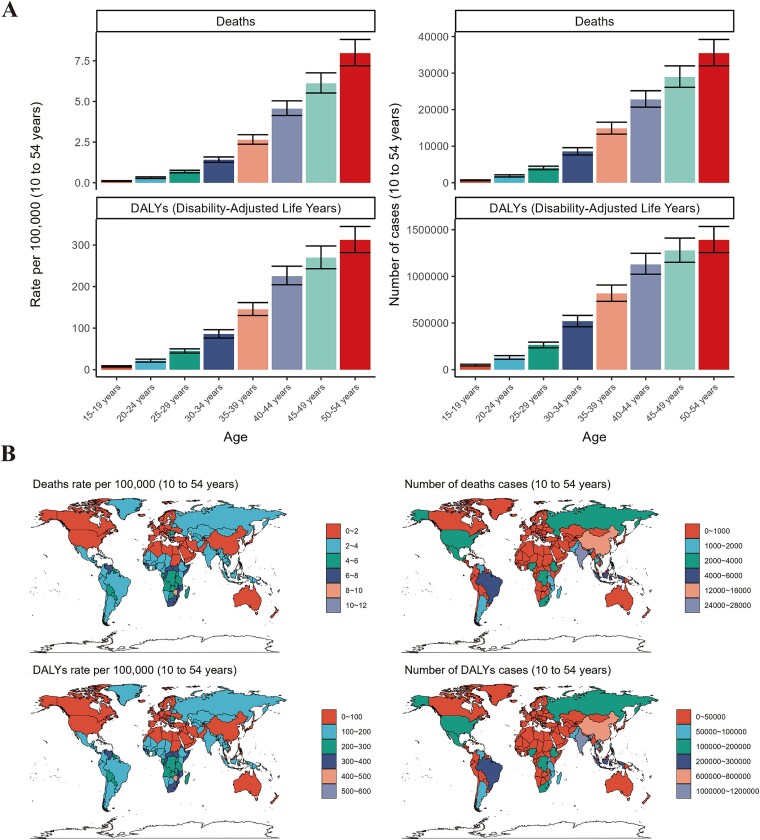
Deaths and DALYs of CC associated with sexual transmission of HPV in subgroups in people aged 10-54 years in 2021. (A) Age subgroup and (B) country subgroup. Abbreviations: CC, cervical cancer; DALYs, disability-adjusted life years; HPV, human papillomavirus.

**Figure 2:**
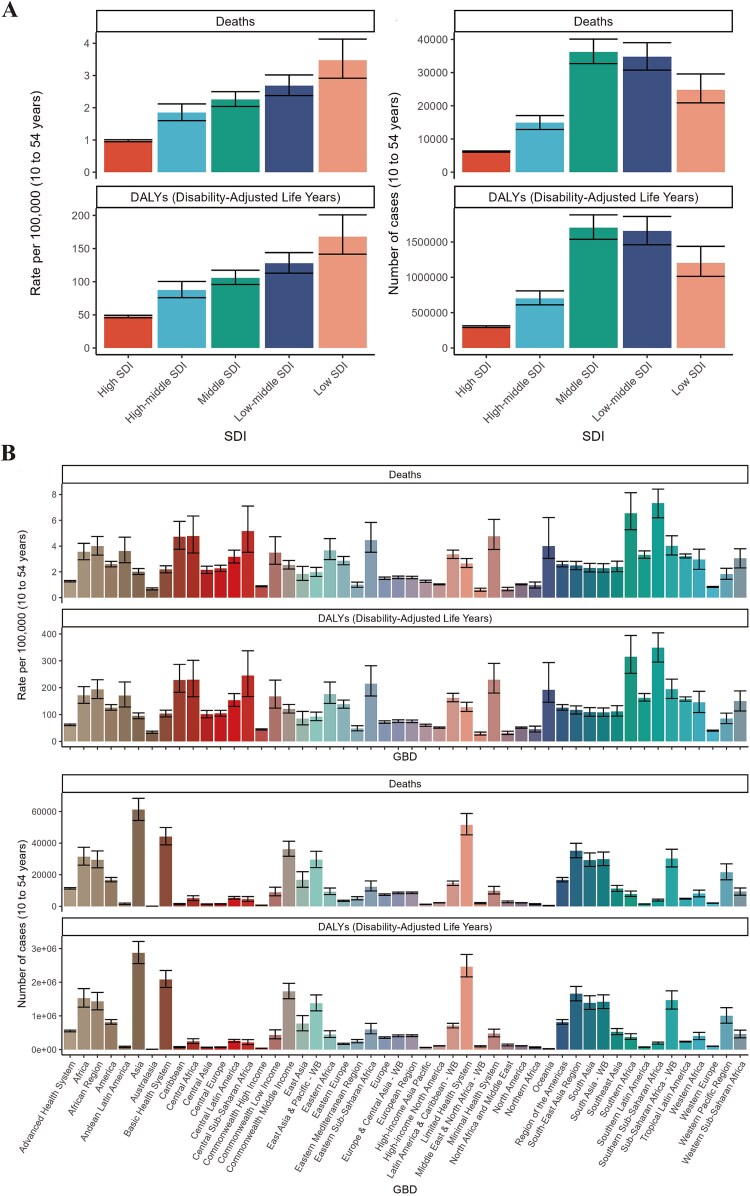
Deaths and DALYs of CC associated with sexual transmission of HPV in subgroups in people aged 10-54 years in 2021. (A) SDI subgroup and (B) GBD subgroup. Abbreviations: CC, cervical cancer; DALYs, disability-adjusted life years; GBD, global burden of disease; HPV, human papillomavirus; SDI, Sociodemographic Index.

In 2021, the global number of CC deaths associated with sexual transmission of HPV among 10-54 year olds was 117 060 (95% UI, 106 690-128 467), and the corresponding ASDR was 2.31 (95% UI, 2.11-2.54)/100 000. The number of DALYs was 5 572 161 (95% UI, 5 057 538-6 116 191), and the corresponding age-standardized DALYs rate was 110.12 (95% UI, 99.95-120.87)/100 000 (Tables 1 and 2). From the perspective of age, the burden of disease gradually increases with the increase of age (Figure 1A). At the national level, the country with the highest number of deaths (24 241 [95% UI, 20 460-28 275]) and the highest number of DALYs (1 142 403 [95% UI, 965 403-1 331 993]) was India, followed by China, Indonesia, Brazil, and Ethiopia. ASDR (11.58 [95% UI, 8.04-16.7]/100 000) and age-standardized DALYs rate (554.25 [95% UI, 382.3-797.76]/100 000) were highest in Kiribati (Figure 1B). In terms of the SDI region, the middle SDI region had the highest number of CC-related deaths and number of DALYs. In low SDI areas, ASDR and age-standardized DALYs rates were highest, at 3.47 (95% UI, 2.92-4.13)/100 000 and 167.94 (95% UI, 141.53-200.76)/100 000, respectively. And in high SDI, ASDR (0.97 [95% UI, 0.94-1.01]/100 000) and age-standardized DALYs rate (47.23 [95% UI, 45.55-49.36]) were the lowest (Figure 2A). The disease burden of CC varies widely across different regions of the globe, with the highest ASDR and age-standardized DALYs rates in Southern sub-Saharan Africa at 7.33 (95% UI, 6.2-8.42)/100 000 and 349.69 (95% UI, 295.2-404.08)/100 000, followed by Southern Africa. The lowest disease burden was found in the Middle East and North Africa-WB region, with an ASDR of 0.61 (95% UI, 0.5-0.74)/100 000, and the age-standardized DALY rates was only 28.82 (95% UI, 23.63-34.75)/100 000 (Figure 2B).

**Table 1:** Number of deaths and age-standardized death rates of CC associated with sexual transmission of HPV among early-aged and middle-aged 10-54 years in 1990 and 2021 and EAPCs from 1990 to 2021.

Characteristics	Number of deaths cases (95% UI) in 1990	The ASDR/100 000 (95% UI) in 1990	Number of deaths cases (95% UI) in 2021	The ASDR/100 000 (95% UI) in 2021	EAPC (95% CI)
**Global**	94 512 (86 198, 103 552)	2.73 (2.49, 2.99)	117 060 (106 690, 128 467)	2.31 (2.11, 2.54)	-0.5 (-0.58, -0.41)
**Age**					
15-19 years	699 (601 798)	0.13 (0.12, 0.15)	642 (538 781)	0.1 (0.09, 0.13)	-1.04 (-1.13, -0.95)
20-24 years	1764 (1548, 2046)	0.36 (0.31, 0.42)	1834 (1573, 2154)	0.31 (0.26, 0.36)	-0.75 (-0.91, -0.59)
25-29 years	3923 (3476, 4389)	0.89 (0.79, 0.99)	4060 (3576, 4524)	0.69 (0.61, 0.77)	-0.86 (-0.98, -0.73)
30-34 years	7756 (7031, 8661)	2.01 (1.82, 2.25)	8557 (7590, 9581)	1.42 (1.26, 1.59)	-1.17 (-1.22, -1.12)
35-39 years	13 550 (12 240, 14 826)	3.85 (3.47, 4.21)	14 815 (13 289, 16 556)	2.64 (2.37, 2.95)	-1.24 (-1.32, -1.15)
40-44 years	18 968 (17 189, 20 953)	6.62 (6, 7.31)	22 794 (20 680, 25 181)	4.56 (4.13, 5.03)	-1.28 (-1.42, -1.15)
45-49 years	21 621 (19 739, 23 914)	9.31 (8.5, 10.3)	28 917 (26 124, 31 987)	6.11 (5.52, 6.76)	-1.38 (-1.5, -1.25)
50-54 years	26 230 (23 645, 28 886)	12.34 (11.12, 13.59)	35 442 (32 009, 39 196)	7.97 (7.19, 8.81)	-1.38 (-1.55, -1.22)
**SDI region**					
High-middle SDI	13 541 (12 388, 14 805)	1.92 (1.75, 2.09)	14 924 (12 869, 17 053)	1.85 (1.6, 2.12)	0.09 (-0.02, 0.2)
High SDI	8783 (8599, 8974)	1.54 (1.51, 1.58)	6190 (6013, 6403)	0.97 (0.94, 1.01)	-1.49 (-1.53, -1.45)
Low-middle SDI	27 009 (23 154, 30 907)	3.72 (3.18, 4.25)	34 765 (30 760, 39 027)	2.69 (2.38, 3.02)	-0.94 (-1.12, -0.76)
Low SDI	16 706 (14 042, 20 197)	5.62 (4.72, 6.79)	24 853 (20 891, 29 599)	3.47 (2.92, 4.13)	-1.76 (-1.89, -1.63)
Middle SDI	28 354 (25 827, 31 072)	2.45 (2.24, 2.69)	36 211 (32 724, 40 118)	2.26 (2.04, 2.5)	-0.18 (-0.26, -0.1)
**GBD region**					
Advanced health system	14 907 (14 554, 15 213)	1.76 (1.72, 1.8)	11 404 (10 921, 11 867)	1.28 (1.22, 1.33)	-1.06 (-1.15, -0.97)
Africa	16 747 (14 222, 19 877)	4.38 (3.72, 5.2)	31 538 (26 109, 37 418)	3.55 (2.94, 4.21)	-0.68 (-0.71, -0.64)
African region	15 650 (13 227, 18 623)	5.13 (4.33, 6.1)	29 490 (24 396, 35 041)	3.99 (3.3, 4.74)	-0.81 (-0.84, -0.77)
America	13 300 (12 997, 13 626)	2.86 (2.79, 2.93)	16 764 (15 482, 18 253)	2.59 (2.39, 2.82)	-0.53 (-0.64, -0.42)
Andean Latin America	1085 (952, 1248)	4.45 (3.9, 5.11)	1589 (1191, 2064)	3.61 (2.71, 4.69)	-0.94 (-1.11, -0.77)
Asia	54 005 (47 379, 60 735)	2.57 (2.26, 2.89)	61 194 (54 340, 68 375)	2.02 (1.8, 2.26)	-0.63 (-0.78, -0.49)
Australasia	223 (206, 242)	1.68 (1.55, 1.82)	123 (109, 139)	0.67 (0.6, 0.76)	-2.62 (-2.84, -2.4)
Basic health system	33 564 (30 082, 37 465)	2.16 (1.94, 2.41)	44 168 (38 882, 49 899)	2.19 (1.93, 2.47)	0.21 (0.13, 0.29)
Caribbean	1095 (950, 1262)	4.73 (4.1, 5.45)	1438 (1144, 1804)	4.72 (3.75, 5.92)	0.02 (-0.04, 0.09)
Central Africa	2369 (1858, 2980)	6.04 (4.73, 7.6)	5076 (3670, 6713)	4.79 (3.46, 6.33)	-0.78 (-0.81, -0.75)
Central Asia	1041 (984, 1106)	2.39 (2.26, 2.54)	1336 (1156, 1511)	2.16 (1.87, 2.44)	0.15 (-0.06, 0.36)
Central Europe	2906 (2777, 3026)	3.65 (3.49, 3.8)	1517 (1353, 1681)	2.27 (2.02, 2.52)	-1.83 (-2.17, -1.5)
Central Latin America	4288 (4175, 4405)	4.02 (3.92, 4.13)	5331 (4531, 6210)	3.16 (2.69, 3.68)	-1.07 (-1.23, -0.91)
Central sub-Saharan Africa	2014 (1479, 2662)	6.17 (4.53, 8.15)	4488 (3062, 6177)	5.16 (3.52, 7.11)	-0.53 (-0.58, -0.48)

Abbreviations: ASDR, age-standardized death rates; CC, cervical cancer; EAPC, estimated annual percentage change; GBD, global burden of disease; HPV, human papillomavirus; SDI, Sociodemographic Index; UI, uncertainty interval.

**Table 2:** Number of DALYs and age-standardized DALYs rates of CC associated with sexual transmission of HPV among early aged and middle-aged 10-54 years in 1990 and 2021 and EAPCs from 1990 to 2021.

Characteristics	Number of DALYs cases in millions (95% UI) in 1990	The age-standardized DALYs rate/100 000 (95% UI) in 1990	Number of DALYs cases in millions (95% UI) in 2021	The age-standardized DALYs rate/100 000 (95% UI) in 2021	EAPC (95% CI)
**Global**	4.53 (4.14, 4.96)	130.92 (119.72, 143.52)	5.57 (5.06, 6.12)	110.12 (99.95, 120.87)	-0.54 (-0.62, -0.47)
**Age**					
15-19 years	0.0518 (0.0446, 0.0595)	9.97 (8.58, 11.45)	0.0480 (0.0402, 0.0583)	7.69 (6.44, 9.34)	-1.02 (-1.1, -0.93)
20-24 years	0.122 (0.107, 0.142)	24.85 (21.77, 28.9)	0.128 (0.110, 0.150)	21.42 (18.38, 25.18)	-0.73 (-0.88, -0.57)
25-29 years	0.255 (0.226, 0.285)	57.54 (51.08, 64.34)	0.266 (0.235, 0.296)	45.14 (39.88, 50.23)	-0.83 (-0.95, -0.7)
30-34 years	0.465 (0.419, 0.514)	120.57 (108.79, 133.41)	0.518 (0.460, 0.582)	85.76 (76.05, 96.26)	-1.13 (-1.18, -1.08)
35-39 years	0.738 (0.665, 0.809)	209.43 (188.79, 229.54)	0.816 (0.732, 0.907)	145.41 (130.56, 161.7)	-1.2 (-1.29, -1.12)
40-44 years	0.930 (0.843, 1.03)	324.73 (294.29, 358.42)	1.13 (1.02, 1.25)	225.55 (204.47, 249.23)	-1.25 (-1.38, -1.12)
45-49 years	0.947 (0.868, 1.05)	407.94 (373.62, 450.62)	1.28 (1.15, 1.41)	270.21 (243.16, 297.91)	-1.35 (-1.47, -1.22)
50-54 years	1.02 (0.917, 1.12)	479.53 (431.59, 525.29)	1.39 (1.25, 1.53)	312.18 (282.03, 344.91)	-1.36 (-1.52, -1.19)
**SDI region**					
High-middle SDI	0.648 (0.592, 0.708)	91.64 (83.77, 100.14)	0.704 (0.611, 0.808)	87.43 (75.88, 100.37)	0.02 (-0.09, 0.12)
High SDI	0.436 (0.424, 0.450)	76.69 (74.58, 79.05)	0.301 (0.290, 0.314)	47.23 (45.55, 49.36)	-1.57 (-1.61, -1.53)
Low-middle SDI	1.28 (1.10, 1.46)	176.28 (151.6, 201.17)	1.66 (1.46, 1.86)	128.1 (112.89, 143.84)	-0.95 (-1.11, -0.8)
Low SDI	0.795 (0.668, 0.962)	267.23 (224.41, 323.45)	1.20 (1.01, 1.44)	167.94 (141.53, 200.76)	-1.71 (-1.82, -1.59)
Middle SDI	1.36 (1.24, 1.49)	117.87 (107.21, 128.93)	1.70 (1.54, 1.88)	106.06 (95.79, 117.33)	-0.28 (-0.35, -0.22)
**GBD region**					
Advanced health system	0.727 (0.708, 0.745)	85.94 (83.75, 88.03)	0.551 (0.526, 0.578)	61.6 (58.81, 64.63)	-1.11 (-1.18, -1.03)
Africa	0.808 (0.685, 0.962)	211.55 (179.27, 251.84)	1.53 (1.26, 1.81)	171.76 (141.85, 203.88)	-0.69 (-0.73, -0.65)
African region	0.756 (0.637, 0.899)	247.55 (208.78, 294.53)	1.43 (1.18, 1.70)	193.34 (159.51, 229.73)	-0.82 (-0.86, -0.77)
America	0.654 (0.637, 0.671)	140.6 (136.88, 144.27)	0.817 (0.753, 0.888)	125.97 (116.2, 137.03)	-0.57 (-0.69, -0.44)
Andean Latin America	0.0520 (0.0457, 0.0595)	213.05 (187.34, 243.63)	0.0751 (0.0564, 0.0973)	170.79 (128.23, 221.28)	-0.99 (-1.16, -0.82)
Asia	2.56 (2.25, 2.88)	122.12 (107, 137.32)	2.87 (2.55, 3.21)	94.79 (84.34, 106.06)	-0.7 (-0.82, -0.58)
Australasia	0.0113 (0.0104, 0.0123)	84.94 (78.16, 92.6)	0.00623 (0.00554, 0.00704)	33.92 (30.16, 38.33)	-2.61 (-2.85, -2.37)
Basic health system	1.62 (1.46, 1.81)	104.29 (93.85, 116.6)	2.08 (1.84, 2.35)	103.05 (91.28, 116.39)	0.1 (0.02, 0.17)
Caribbean	0.0538 (0.0466, 0.0622)	232.39 (201.31, 268.82)	0.0698 (0.0557, 0.0874)	228.89 (182.83, 286.78)	-0.04 (-0.12, 0.03)
Central Africa	0.113 (0.0887, 0.142)	286.94 (226.18, 361.79)	0.244 (0.176, 0.320)	229.77 (166.22, 301.92)	-0.77 (-0.8, -0.73)
Central Asia	0.0500 (0.0473, 0.0531)	114.79 (108.61, 121.91)	0.0625 (0.0542, 0.0713)	101.14 (87.71, 115.35)	-0.03 (-0.24, 0.17)
Central Europe	0.139 (0.133, 0.145)	174.98 (166.77, 182.42)	0.0700 (0.0628, 0.0774)	104.75 (93.97, 115.78)	-1.95 (-2.25, -1.65)
Central Latin America	0.209 (0.203, 0.215)	196.05 (190.5, 201.38)	0.259 (0.220, 0.300)	153.58 (130.36, 178.11)	-1.09 (-1.26, -0.92)
Central sub-Saharan Africa	0.0952 (0.0702, 0.126)	291.51 (214.85, 386.53)	0.213 (0.145, 0.293)	245.63 (166.75, 337.51)	-0.53 (-0.58, -0.48)

Abbreviations: CC, cervical cancer; DALYs, disability-adjusted life years; EAPC, estimated annual percentage change; GBD, global burden of disease; HPV, human papillomavirus; SDI, Sociodemographic Index; UI, uncertainty interval.

### Changes in the global burden of cervical cancer associated with sexual transmission of human papillomavirus in people aged 10-54 years from 1990 to 2021

**Figure 3:**
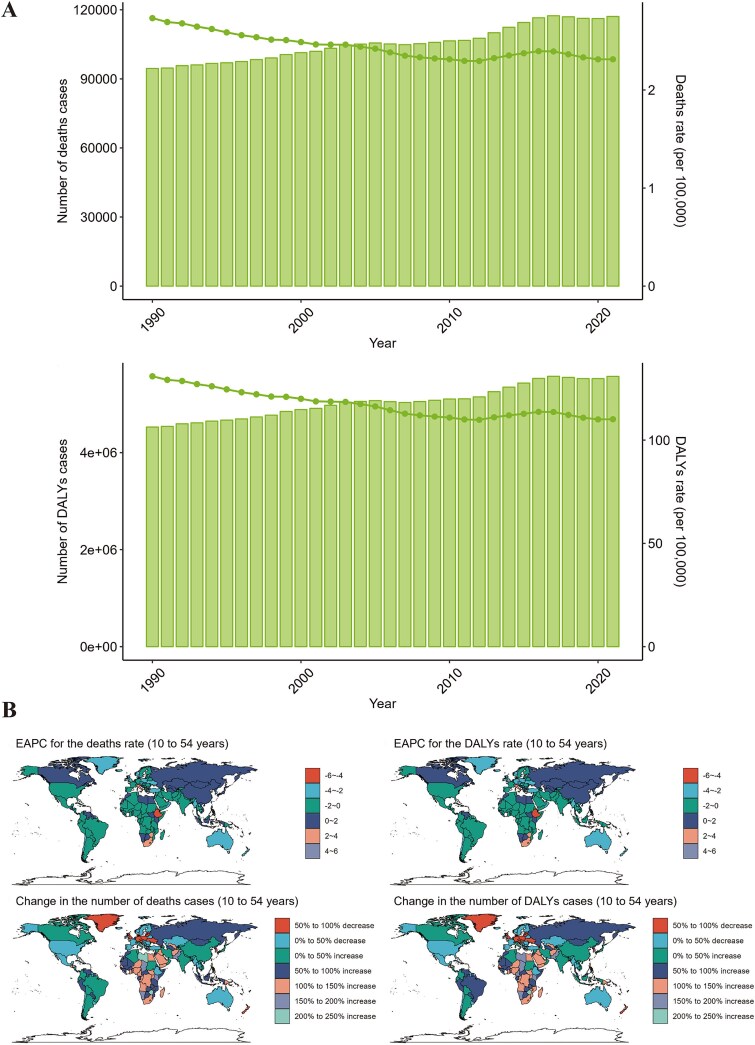
Trends in CC deaths and DALYs associated with sexual transmission of HPV in people aged 10-54 years from 1990 to 2021. (A) Overall trend of change and (B) EAPC changes in different countries. Abbreviations: CC, cervical cancer; DALYs, disability-adjusted life years; EAPC, estimated annual percentage change; HPV, human papillomavirus.

**Figure 4:**
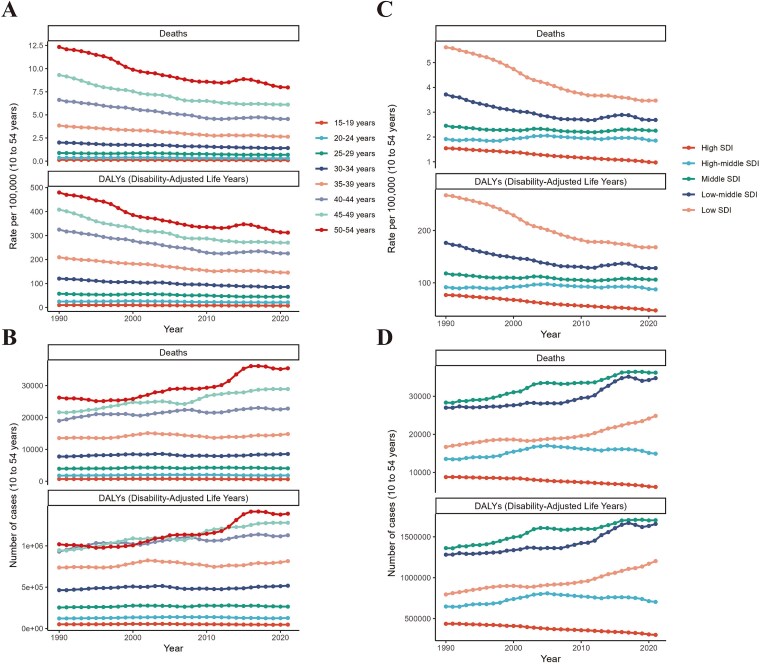
Trends in CC deaths and DALYs associated with sexual transmission of HPV in people aged 10-54 years from 1990 to 2021. (A) Trends in the number of deaths and DALYs in age subgroups, (B) trends in ASDR and age-standardized DALYs rates in age subgroups, (C) trends in the number of deaths and DALYs in SDI subgroups, and (D) trends in ASDR and age-standardized DALYs rates in SDI subgroups. Abbreviations: ASDR, age-standardized death rates; CC, cervical cancer; DALYs, disability-adjusted life years; HPV, human papillomavirus; SDI, Sociodemographic Index.

**Figure 5:**
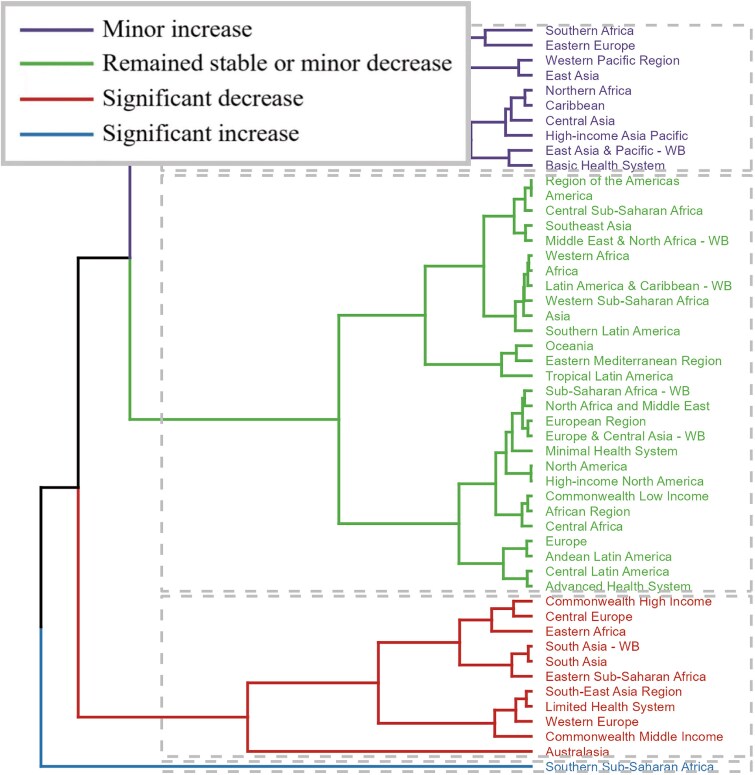
Cluster analysis of trends in the disease burden of CC in people aged 10-54 years from 50 regions around the world. Abbreviations: CC, cervical cancer.

Between 1990 and 2021, the number of CC deaths associated with sexual transmission of HPV increased by 23.9% and the number of DALYs increased by 23.1% globally in people aged 10-54 years, while the corresponding ASDR (EAPC: -0.5 [95% CI, -0.58 to 0.41]) and age-standardized DALYs rate (EAPC: -0.54 [95% CI, -0.62 to -0.47]) showed significant downward trends (Figure 3A). The ASDR decreased from 2.73 (95% UI, 2.49-2.99)/100 000 in 1990 to 2.31 (95% UI, 2.11-2.54)/100 000 in 2021, while the age-standardized DALYs rate decreased from 130.92 (95% UI, 119.72-143.52)/100 000 to 110.12 (95% UI, 99.95-120.87)/100 000. In terms of countries, the top 3 countries with the largest increases in the number of CC-related deaths were the United Arab Emirates, Zimbabwe, and Gambia, all more than twice. Lesotho and Zimbabwe saw the fastest increase in ASDR and age-standardized DALYs rates, while Maldives and Eswatini saw the most dramatic decline (Figure 3B). In terms of age, the disease burden of CC in the under-40 age-group has remained basically stable. Although the number of deaths and the number of DALYs increased in the 40-54 age-group compared with 1990, the corresponding ASRs showed a significant downward trend (Figure 4A and B). In terms of the SDI region, the number of CC-related deaths and the number of DALYs in the middle SDI region has always occupied the first place. CC-related ASDR and age-standardized DALYs rate decreased most significantly in low SDI areas, followed by high SDI areas, and increased slightly in high-middle SDI areas (Figure 4C and D). Cluster analysis results showed that the burden of CC-related disease was classified as significantly increasing in Southern sub-Saharan Africa. Regions such as Commonwealth High Income, Central Europe, and Eastern Africa were in the significant decline category. In addition, regions such as Southern Africa and Eastern Europe were in the category of slight increases, while the Region of the Americas and Central sub-Saharan Africa were in the category of stable or slight declines (Figure 5).

### Frontier analysis of the association between cervical cancer disease burden associated with sexual transmission of human papillomavirus and Sociodemographic Index in people aged 10-54 years

**Figure 6:**
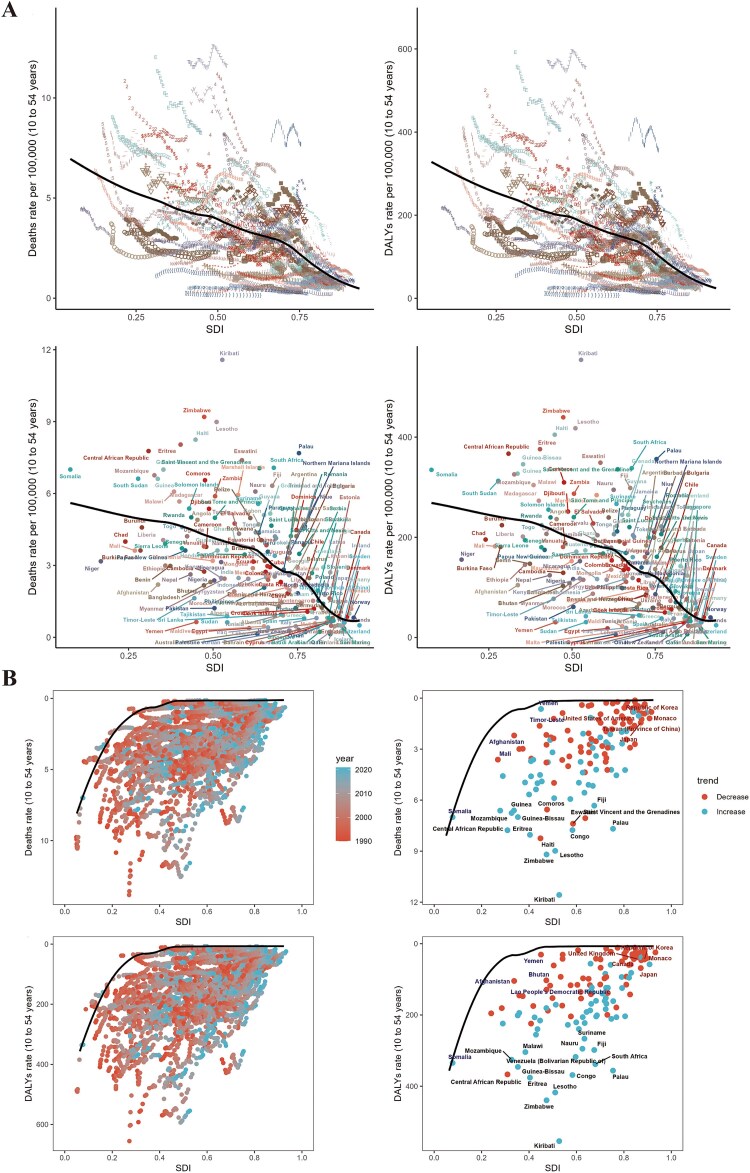
Frontier analysis of the association between CC disease burden-associated with sexual transmission of HPV and SDI in people aged 10-54 years. (A) Association between the number of CC deaths or DALYs attributable to sexual transmission of HPV and SDI among individuals aged 10-54 years in different GBD regions worldwide from 1990 to 2021. (B) Frontier analysis with ASDR and age-standardized DALYs rates. The frontier is marked using a solid black color, and countries and territories are presented as dots. The leading 15 countries with the most EF are marked in black. Among low SDI countries, those closest to the frontier line are represented as having the smallest EF relative to their level of development and are marked in blue, indicating that these countries have better disease control for CC associated with sexual transmission of HPV. Among high SDI countries, those farthest from the frontier line show the largest EF and are marked in red. The larger the difference in effectiveness, the more room for improvement in the control of CC in the country. Abbreviations: ASDR, age-standardized death rates; CC, cervical cancer; DALYs, disability-adjusted life years; EF, effective difference; GBD, global burden of disease; HPV, human papillomavirus; SDI, Sociodemographic Index.

Our study shows that the burden of CC associated with sexual transmission of HPV in people aged 10-54 is inversely associated with SDI. With the rise of SDI, the burden of CC-related disease decreased significantly (Figure 6A). In order to explore the ideal situation in which countries can control the disease burden under the corresponding SDI conditions, we conducted a frontier analysis, and the results of the frontier analysis showed that the disease burden and effective differences between different countries gradually decreased as SDI increased. Among low SDI countries, those closest to the frontier line are represented as having the smallest effective difference relative to their level of development and are marked in blue, indicating that these countries have better disease control for CC associated with sexual transmission of HPV. Among high SDI countries, those farthest from the frontier line show the largest effective difference and are marked in red. The 15 countries with the largest effective differences are marked in black. The larger the difference in effectiveness, the more room for improvement in the control of CC in the country. For the CC-related death rate, the 5 low-SDI countries closest to the frontier line are Somalia, Yemen, Mali, Afghanistan, and Timor-Leste. The countries with the greatest difference in effectiveness include Kiribati, Zimbabwe, Lesotho, Haiti, Congo, and others. The 5 countries closest to the frontier line in terms of CC-related DALY rates are Somalia, Afghanistan, Yemen, Bhutan, and Lao People’s Democratic Republic. The 5 countries furthest from the frontier line include the United Kingdom, Republic of Korea, Canada, Monaco, and Japan. Countries with the greatest differences in effectiveness include Zimbabwe, Lesotho, Eritrea, Central African Republic, Congo, and others (Figure 6B).

### Influencing factors of estimated annual percentage change

**Figure 7:**
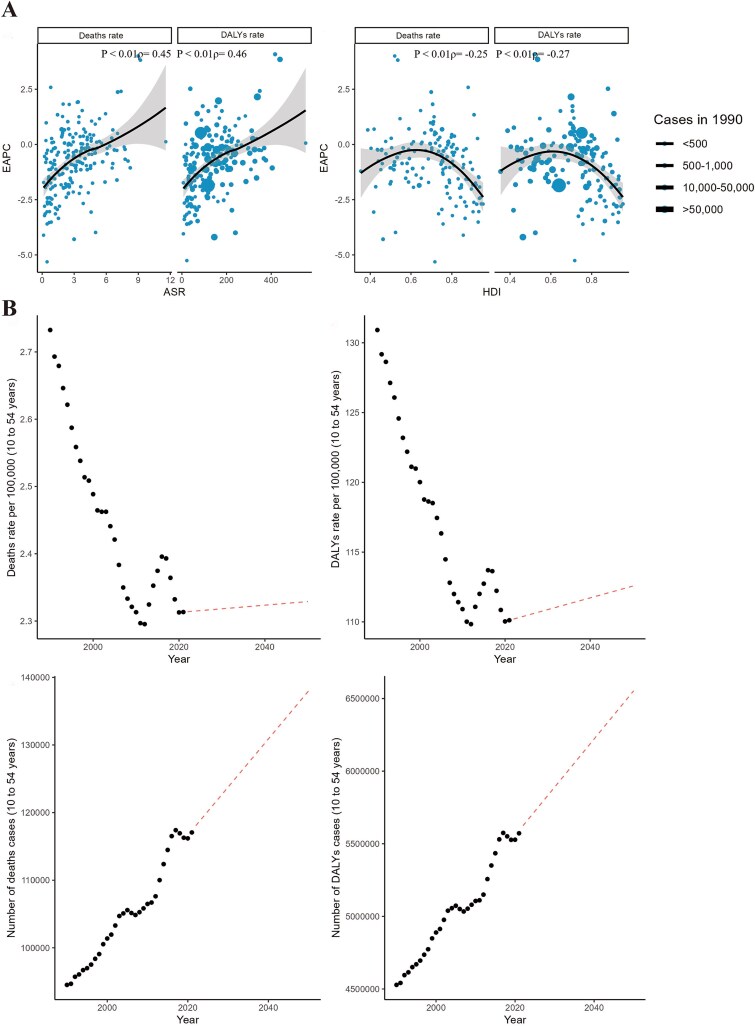
(A) Influencing factors of EAPC and (B) the projection of CC burden associated with sexual transmission of HPV among individuals aged 10-54 years from 2022 to 2050 includes the estimated number of deaths or DALYs and the corresponding ASRs. Abbreviations: ASR, age-standardized rate; CC, cervical cancer; DALYs, disability-adjusted life years; EAPC, estimated annual percentage change; HPV, human papillomavirus.

To examine the relationship between EAPC since 1990 and ASR and the Human Development Index (HDI) for 2021, we used the 1990 CC-associated ASR associated with sexual transmission of HPV as a baseline measure of disease burden. The 2021 HDI is a measure of access and quality of healthcare across countries. Our analysis revealed a positive correlation between EAPC and ASR: as ASR increases, EAPC gradually increases, specifically, the correlation is as follows: ASDR (ρ = 0.45, *P* < .01) and age-standardized DALY rate (ρ = 0.46, *P* < .01). In addition, our results show a subtle relationship between EAPC and HDI: when HDI <0.6, EAPC and HDI are positively correlated, and when HDI >0.6, EAPC and HDI are negatively correlated. The death rate (ρ = -0.25, *P* < .01) and DALY rate (ρ = -0.27, *P* < .01) showed a similar trend (Figure 7A).

### Prediction of future trends in cervical cancer disease burden associated with sexual transmission of human papillomavirus in people aged 10-54 years

According to the ARIMA model, the global burden of CC associated with sexual transmission of HPV among people aged 10-54 years is projected to increase during the period 2021-2050. It is estimated that by 2050, the associated deaths and number of DALYs will reach 138 019 and 653 427, respectively, with corresponding ASRs of 2.33/100 000 and 112.56/100 000 (Table S1 and Figure 7B).

## Discussion

To our knowledge, this is the first study to describe changes in the burden of CC disease associated with sexual transmission of HPV in people aged 10-54 years from 1990 to 2021 at global, regional, and national levels. Over the past 3 decades, the number of deaths from CC and the number of DALYs associated with sexual transmission of HPV among people aged 10-54 has increased globally, while the ASDR and age-standardized DALY rates have shown a significant downward trend. The burden of disease varies significantly across countries and regions, with the highest number of deaths and number of DALYs in absolute terms in Asia, especially India and China. However, when ASDR and age-standardized DALY rates are considered, they are highest in Southern sub-Saharan Africa and Southern Africa, particularly Zimbabwe and Lesotho. Globally, the burden of CC-related disease shows a significant negative correlation with SDI. CC-related ASDR and age-standardized DALY rates decreased significantly in all SDI regions, except high-middle SDI regions. Although ASDR and age-standardized DALY rates were highest in low SDI areas, the decline in ASDR and age-standardized DALY rates was also most significant in this region. From 1990 to 2021, the middle SDI region has always occupied the first place in the number of CC-related deaths and the number of DALYs. The burden of CC-related disease in Southern sub-Saharan Africa is classified as significantly increasing. Regions such as Commonwealth High Income, Central Europe, and Eastern Africa were in the significant decline category. In addition, the burden of CC associated with sexual transmission of HPV increases with age in people aged 10-54 years. The ARIMA prediction model shows that the burden will increase over the next 25 years.

The premise was to emphasize that the findings underscore the urgent need for equitable and timely implementation of HPV vaccination programs, particularly in regions where exposure to HPV infection remains high due to structural factors such as limited access to prevention services, rather than focusing on individual-level behavioral judgments.

From a public policy perspective, our findings carry several actionable implications. First, the substantial burden observed in low-SDI regions and sub-Saharan Africa underscores the need for accelerated investment in HPV vaccination infrastructure, including vaccine procurement, cold chain maintenance, and community engagement strategies to address vaccine hesitancy. Second, the persistent burden in middle-SDI regions, particularly in Asia, highlights the importance of integrating screening programs with vaccination efforts, as these populations may have missed earlier vaccination opportunities. Third, the projected increase in burden over the next 25 years calls for sustained political commitment and multi-sectoral collaboration, including health education campaigns that address behavioral risk factors without stigmatizing affected individuals. Policymakers should prioritize gender-neutral vaccination strategies where feasible, as male vaccination can contribute to herd immunity and further reduce HPV transmission. Finally, given the heterogeneity in burden across regions, tailored strategies that account for local epidemiological, cultural, and health system contexts are essential to achieving the World Health Organization (WHO) 90-70-90 elimination targets by 2030.

From 1990 to 2021, the number of deaths and the number of DALYs related to sexual transmission of HPV among people aged 10-54 have increased globally, which may be caused by the increase in the global individual base and the aging of the individual,^14^^,^^15^ the number of cancer cases tends to increase with the growth of age.^16^ In fact, ASDR and age-standardized DALY rates have decreased significantly. In Zhang et al.’s study, which analyzed the individual-wide burden of CC associated with sexual transmission of HPV from 1990 to 2019, they showed that over the past few decades, The ASDR and age-standardized DALYs rate of CC caused by sexually transmitted HPV decreased over time, consistent with the findings of this study, which further enhances the reliability of our study. Notably, they found that the burden of CC associated with sexual transmission of HPV was greatest in the 50-54 age-group,^8^ while the prevalence of certain behavioral risk factors was higher in people under 54 years of age, which brought new challenges to the prevention and control of CC and urgently needed targeted public health interventions to reduce the burden of disease in this individual. Using the latest data of GBD-2021, this study focuses on analyzing the disease burden of CC related to sexual transmission of HPV in 10-54 years old and predicts the future trend, which can provide reference for the development of effective CC prevention and control strategies. The results of this study showed a significant decrease in the ASDR and age-standardized DALY rates of CC, indicating that the prevention and control of CC associated with sexual transmission of HPV has achieved some success globally. Data from a study in Spain showed that the prevalence of behavioral patterns such as inconsistent condom use among college students was related to alcohol and marijuana use, and the proportion of women reporting inconsistent condom use under the influence of alcohol was as high as 40.9%.^17^ Another study from Wuhan, China, surveyed 4769 undergraduate students and found that the average age of first sex was only 19.3 (±1.7) years, while women who started sex earlier were less likely to use condoms.^18^ In addition, data from the United States, sub-Saharan Africa, Tanzania, Hong Kong, and other regions show low condom use and high prevalence of multiple sexual partnerships.^7^^,^^19–21^ Thus, the sexual behaviors that influence HPV transmission risk remain a serious challenge for CC prevention and control.

With the popularization of HPV vaccine, the disease burden of CC in people aged 10-54 years has decreased even in the context of high prevalence of these behavioral factors, which fully highlights the important contribution of HPV vaccine in alleviating the burden of CC disease. Since the advent of HPV vaccine in 2006, HPV infection rates and HPV-related high-grade cervical lesions have decreased significantly.^22^^,^^23^ Currently, vaccination has become a strategic intervention measure to curb the burden of this malignant tumor, which brings light to the elimination of CC, a major public health problem. Diaz et al. used a mathematical model to calculate the relative effectiveness and cost-effectiveness of HPV vaccination and CC screening interventions, which is very cost-effective even though vaccination coverage is relatively low, and preadolescent girls are the main target individuals.^24^ At the same time, a global study observed that the proportion of CC caused by HPV types 16 and 18 in women under 40 years old was higher than that in women over 70 years old,^25^ indicating that HPV vaccination in young women is particularly important to reduce the incidence of CC. To provide contextual understanding of the heterogeneous burden patterns observed across regions, we examined regional HPV vaccination coverage based on data from WHO and UNICEF. As of 2022, the global estimated coverage of the final dose of HPV vaccine among girls aged 9-14 years was approximately 21%, with substantial regional disparities: coverage exceeded 70% in high-income countries such as Australia (84%), the United Kingdom (72%), and Sweden (80%), whereas it remained below 10% in most countries in sub-Saharan Africa and South Asia, including Nigeria (6%), Uganda (8%), and India (4%).^26^ In the WHO African Region, which bears the highest age-standardized burden of CC, coverage was estimated at only 15% in 2022, far below the 90% target set by the WHO Global Strategy for Cervical Cancer Elimination.^27^ These stark regional disparities in vaccination uptake closely parallel the burden patterns observed in our study, with the highest mortality and DALY rates concentrated in regions where vaccination coverage remains critically low. As of early 2019, 115 countries and territories (95 of which are WHO Member States) had included HPV vaccines in their national programs.^28^ In addition, in 2020, the World Health Organization adopted the Global Strategy to Eliminate CC, which aims to achieve the 90-70-90 goal of eliminating CC by 2030, reducing and maintaining the incidence of CC at less than 4 per 100 000 people. These targets aim to fully vaccinate 90% of girls by age 15, screen 70% of women by age 35 and again by age 45, and provide treatment to 90% of women diagnosed with cervical disease.^29^ Notably, the European Cancer Organization considers the implementation of gender-neutral vaccination programs as an important health strategy and encourages all EU member States to introduce gender-neutral vaccination programs by 2030. The European Commission’s “European Plan to Beat Cancer” also highlights the importance of increasing HPV vaccination for boys by 2030,^30^ with the potential to further reduce the disease burden of CC associated with sexual transmission of HPV through public health interventions that address the issue of vaccination for both men and women.

We also observed a significant negative correlation between CC-related disease burden and SDI. The burden of CC associated with sexual transmission of HPV is particularly high in sub-Saharan and Southern Africa and is a major challenge to achieving the global goal of eliminating CC. 2019 data show that adolescent girls and young adult women aged 15-24 years account for 24% of new HIV infections in Southern sub-Saharan Africa, more than double their 10% share of the individual, which may be related to their sexual behavior patterns.^31^ The overall prevalence of transactional sex among women in sub-Saharan Africa is 12.55% (9.59%-15.52%),^32^ and condom use among university students in the region is low.^33^ The high prevalence of multiple partnerships and inconsistent condom use in the area promotes the spread of HPV. In addition, HPV vaccination and screening show significant imbalances in different regions. Vaccination against the HPV is available in less than 30% of LMICs, while in high-income countries the proportion is more than 85%. Only about 20% of women in LMICs have been screened for CC, compared with more than 60% in high-income countries.^34^ Compared to the rest of the world, sub-Saharan Africa has made limited progress in the implementation of global HPV vaccination programs, mainly due to the lack of vaccine resources in the region, lack of information about vaccination services, insufficient cold chain capacity, and misconceptions about vaccine side effects and vaccine safety.^35^ A comparative model analysis of 78 low-and lower-middle-income countries by Prof. Marc Brisson and his team projected data showing that girls-only HPV vaccination would reduce the median age-standardized incidence of CC in LMICs from 19.8 to 2.1 cases per 100 000 woman years over the next century (an 89.4% reduction). And avoid 61 million cases during that time. Two additional screenings would reduce the incidence to 0.7 cases per 100 000 women (a 96.7% reduction) and averted an additional 12.1 million cases.^34^ Therefore, HPV vaccination and screening are key links in achieving the strategic goal of eliminating CC.

Our prediction model shows an increase in the burden of sexually transmitted HPV-related CC in 10-54 year olds over the next 25 years, suggesting that the prevention and control of CC remain a serious challenge, as predicted by Simms et al. Globally, 44.4 million cases of CC will be diagnosed during 2020-69, with nearly two-thirds of these cases occurring in low or medium HDI countries. A rapid scale-up of vaccination to 80%-100% global coverage using a broad-spectrum HPV vaccine, starting in 2020, could avert 67-7.7 million cases over that period, but more than half of these cases would be avoided after 2060.^36^ For younger people, addressing behavioral risk factors through health education, increasing HPV vaccination coverage, and providing cervical screening for older people WHO would not benefit from HPV vaccination will help to achieve the WHO triple intervention strategy of 90/70/90 to eliminate CC as early as possible.

We acknowledge the limitation regarding the definition of the risk factor. The GBD database operationalizes the exposure as “sexual behaviors that influence HPV transmission risk” based on a standardized comparative risk assessment framework. However, we recognize that this definition, while necessary for cross-country comparability, cannot capture all local behavioral nuances. We have therefore reframed our language throughout the manuscript to avoid value-laden terms such as “unsafe” and instead use neutral epidemiological descriptors. Future research should aim to develop culturally sensitive metrics that complement global estimates. Other limitations include: the accuracy of our estimates of CC disease burden may be affected by the quality and integrity of the original data. In the 1990s, many countries had less-developed disease registry systems and restricted availability of Internet access, resulting in incomplete registries and missing data, which could lead to an underestimate of the true number of cases. In areas where data are lacking, estimates rely on modeling and historical trends, which can introduce discrepancies and variability. Finally, HPV vaccine coverage data for older populations in many countries are incomplete, which limits our ability to draw direct conclusions about the effectiveness of HPV vaccination against CC. Despite these limitations, our study provides valuable insights into trends in the HPV-associated burden of cervical-related disease in people aged 10-54.

## Conclusion

Although the number of deaths and the number of DALYs associated with sexual transmission of HPV in the 10-54 age-group increased, the corresponding ASDR and age-standardized DALYs decreased significantly, indicating that the global prevention and control of CC have achieved some success. However, the burden of CC varies greatly among different regions, and CC-related disease burden is significantly negatively correlated with SDI, with the most serious disease burden in Southern sub-Saharan Africa and Southern Africa. This highlights the ongoing challenges in addressing the behavioral risk factors that facilitate HPV transmission and increasing HPV vaccination coverage. The projected increase in the burden of CC associated with sexual transmission of HPV in people aged 10-54 by 2050 suggests that the prevention and control of CC remain critical and that effective policies are urgently needed to further increase vaccination coverage among young women, especially in LMICs, and to increase CC screening rates. Raising public awareness of sexual health is essential to the global CC elimination plan.

## Supplementary Material

Table_S1_qfag045

## Data Availability

All data used in this study are freely accessible in GBD 2021 (https://ghdx.healthdata.org/gbd-2021).
