# Supplementary material for: Global, regional, and national burdens of human papillomavirus-associated cervical cancer attributable to sexually acquired human papillomavirus infections among individuals aged 10-54 years from 1990 to 2021: findings from the Global Burden of Disease study 2021
Source: Sex Med. 2026 Jun 16;14(4):qfag045. doi: 10.1093/sexmed/qfag045 (PMC13271409; doi:10.1093/sexmed/qfag045)
Supplement: Table_S1_qfag045 [file table_s1_qfag045.docx]

**Table S1 Prediction of the number of cervical cancer deaths and the number of DALYs and corresponding ASRs associated with unsafe sex in a global population aged 10-54 years**

| **Years** | **The age-standardized deaths rate** **in millions (95% UI) in 1990** | **Number of deaths cases** **in millions (95% UI) in 1990** | **The age-standardized deaths rate/100000 (95% UI) in 2021** | **Number of deaths cases** **in millions (95% UI) in 2021** |
| --- | --- | --- | --- | --- |
| 2022 | 2.31 | 0.118 | 110 | 5.62 |
| 2023 | 2.31 | 0.119 | 110 | 5.66 |
| 2024 | 2.31 | 0.119 | 110 | 5.69 |
| 2025 | 2.32 | 0.120 | 110 | 5.72 |
| 2026 | 2.32 | 0.121 | 111 | 5.76 |
| 2027 | 2.32 | 0.122 | 111 | 5.79 |
| 2028 | 2.32 | 0.122 | 111 | 5.82 |
| 2029 | 2.32 | 0.123 | 111 | 5.86 |
| 2030 | 2.32 | 0.124 | 111 | 5.89 |
| 2031 | 2.32 | 0.124 | 111 | 5.92 |
| 2032 | 2.32 | 0.125 | 111 | 5.96 |
| 2033 | 2.32 | 0.126 | 111 | 5.99 |
| 2034 | 2.32 | 0.127 | 111 | 6.02 |
| 2035 | 2.32 | 0.127 | 111 | 6.06 |
| 2036 | 2.32 | 0.128 | 111 | 6.09 |
| 2037 | 2.32 | 0.129 | 111 | 6.12 |
| 2038 | 2.32 | 0.129 | 112 | 6.16 |
| 2039 | 2.32 | 0.130 | 112 | 6.19 |
| 2040 | 2.32 | 0.131 | 112 | 6.22 |
| 2041 | 2.32 | 0.132 | 112 | 6.25 |
| 2042 | 2.32 | 0.132 | 112 | 6.29 |
| 2043 | 2.33 | 0.133 | 112 | 6.32 |
| 2044 | 2.33 | 0.134 | 112 | 6.35 |
| 2045 | 2.33 | 0.134 | 112 | 6.39 |
| 2046 | 2.33 | 0.135 | 112 | 6.42 |
| 2047 | 2.33 | 0.136 | 112 | 6.45 |
| 2048 | 2.33 | 0.137 | 112 | 6.49 |
| 2049 | 2.33 | 0.137 | 112 | 6.52 |
| 2050 | 2.33 | 0.138 | 113 | 6.55 |
